# Strictly conformal transformation optics for directivity enhancement and unidirectional cloaking of a cylindrical wire antenna

**DOI:** 10.1038/s41598-022-20503-1

**Published:** 2022-09-29

**Authors:** Hossein Eskandari

**Affiliations:** Department of Electrical Engineering, Ferdowsi University of Mashhad, 9177948944 Mashhad, Iran

**Keywords:** Electrical and electronic engineering, Transformation optics

## Abstract

Using conformal transformation optics, a cylindrical shell made of an isotropic refractive index material is designed to improve the directivity of a wire antenna while making it unidirectionally invisible. If the incident wave comes from a specific direction, it is guided around the wire. Furthermore, when an electrical current is used to excite the wire, the dielectric shell transforms the radiated wave into two lateral beams, improving directivity. The refractive index of the dielectric shell is calculated using the transformation optics recipe after establishing a closed-form conformal mapping between an annulus and a circle with a slit. The refractive index is then modified and discretized using a hexagonal lattice. Ray-tracing and full-wave simulations with COMSOL Multiphysics are used to validate the functionality of the proposed shell.

## Introduction

Since Leonhardt and Pendry discovered a transformation-optical solution for the problem of invisibility^1,2^, the theory of transformation optics (TO) has caught the interest of scientists. TO recipe incorporates a geometrical interpretation of the device’s function into a calculated material. This theory allowed researchers to derive the material that could realize a specific functionality, essentially an inverse problem. Many fabulous devices have been created using this theory like field rotators^3–5^, field concentrators^6,7^, carpet cloaks^8–16^, polarization splitters^4,17–20^, beam expanders and squeezers^21,22^, directivity enhancers^23–29^, and waveguide couplers^30–37^. TO has also been used for compressing lenses^38–43^.

Despite being an interesting systematic approach and providing a mathematical path for the design of functional devices, the material calculated by TO is often difficult to realize. In general, the constitutive parameters can possess extreme permittivity and permeability values and a high degree of anisotropy. Attaining anisotropy is quite challenging since the available natural materials exhibit low anisotropy levels. Metamaterial unit cells can provide higher anisotropy at the expense of introducing loss and limiting the bandwidth. The type of transformation used has a significant impact on material complexity. For instance, linear transformations result in a homogeneous anisotropic medium^12,15,18,19,30,31,33,44,45^, whereas using quasi-conformal and conformal transformations minimizes and eliminates the anisotropy, respectively^1,8,23–29,35,41,46–48^.

For the case of an omnidirectional cloak, the medium possesses singular constitutive parameters near the concealment region^2^. The singularity embedded in omnidirectional cloaks stems from using a point-transformed mapping in 3D and a line-transformed mapping in 2D. As a result, a new class of unidirectional invisibility cloaks was introduced, capable of providing invisibility for one incident angle. The mapping is plane-transformed for such unidirectional cloaks, which can lead to material singularity mitigation. Unidirectional cloaks can be designed using linear transformations^49–52^, non-linear transformations^49,53,54^, and conformal transformations^1,55–60^. A cloak prototype was fabricated using metamaterial cells^51^ and metal 3D printing^52^. Employing metasurfaces for the realization of the structure is also an active area of research^61,62^.

On the other hand, conformal and quasi-conformal transformations have been proven to help improve the directivity of a radiating source. Researchers have demonstrated that they can flatten the phase fronts of the cylindrical wave emanating from a point source by using (quasi-) conformal mappings^23–26^. Following a similar approach, a conformal transformation was numerically calculated to minimize the phase error at the horn antenna aperture, leading to a directive beam^27,28,63^.

An interesting TO application is the case of a bi-functional device that provides both unidirectional cloaking and directivity enhancement. Recently, an anisotropic solution was proposed by numerically solving Laplace’s equation^64^. The designed metamaterial shell could cloak the wire antenna for an incident TM wave and enhance the directivity of the wire antenna that emanated TE waves. The same research group recently proposed an isotropic solution using numerical quasi-conformal transformation^65^. The resulting isotropic magnetic transformation medium could be used to unidirectionally cloak the loop antenna in the presence of an incident TM wave and to enhance the directivity of the antenna. Since the incident wave and the radiating wave from the loop antenna were TM polarized, an isotropic magnetic material was used. The magnetic medium was realized using printed SRR and meander line structures, limiting the bandwidth.

The most well-known method for deriving the quasi-conformal mapping between physical and virtual spaces is to solve Laplace’s equation with proper Dirichlet and Neumann boundary conditions. The level of anisotropy neglected by pursuing this method is proportional to the conformal module mismatch between the two spaces. If the difference between the conformal modules of two spaces is significant, the transformation calculated by numerically solving Laplace’s equation will deviate from being quasi-conformal. In such cases, using the strictly conformal TO formula is questionable, both mathematically and physically. In the case of carpet cloaks, for example, even a minor conformal module mismatch has been shown to cause a lateral shift^11^. For the device providing unidirectional cloaking and directivity enhancement, the boundary condition associated with the numerical method procedure does not lead to conformal module matching^65^. As the radius of the concealment region increases, the conformal module mismatch increases, and numerical calculation alone can not remedy this defect. A strictly conformal transformation can fix this issue, leading to a purely conformal solution.

A closed-form, strictly conformal map between the doubly connected physical and virtual spaces is established here, leading to a perfect conformal module match. The resulting isotropic dielectric shell cloaks the wire antenna from an impinging TM wave and enhances the wire antennas’s directivity. Unidirectional invisibility is obtained by keeping the outer boundary of virtual and physical space alike and mapping the inner cylindrical wire to a slit. The latter also bilaterally improves the directivity. The refractive index is then modified and discretized to investigate the practicality of the device. The device’s unidirectional cloaking property is discussed from two distinct perspectives in the “Discussion” section. Ray-tracing and full-wave simulations are carried out using COMSOL to confirm the device’s functionality.

## Design method

Here, the virtual and physical spaces are related to each other by a conformal map. The virtual and physical spaces are denoted by the *w*-plane, which has Cartesian coordinates of (*u*, *v*), and the *z*-plane, which has Cartesian coordinates of (*x*, *y*). Assuming the complex variables $w=u+\mathrm{i}v$ and $z=x+\mathrm{i}y$, the conformal mapping from the physical space to the virtual space is expressed by the analytic function $w=f(z)$. In such a scenario, if the virtual space is filled with the refractive index of *n*(*u*, *v*), the refractive index of the physical space is derived using the following well-known TO formula^1^:

1 $$\begin{aligned} n(x,y) = {\left| {f'(z)} \right| }n(u,v), \end{aligned}$$

where *n*(*u*, *v*) is the refractive index of the virtual space. Here, the refractive index of $n(u,v)=1$ is assumed.

**Figure 1:**
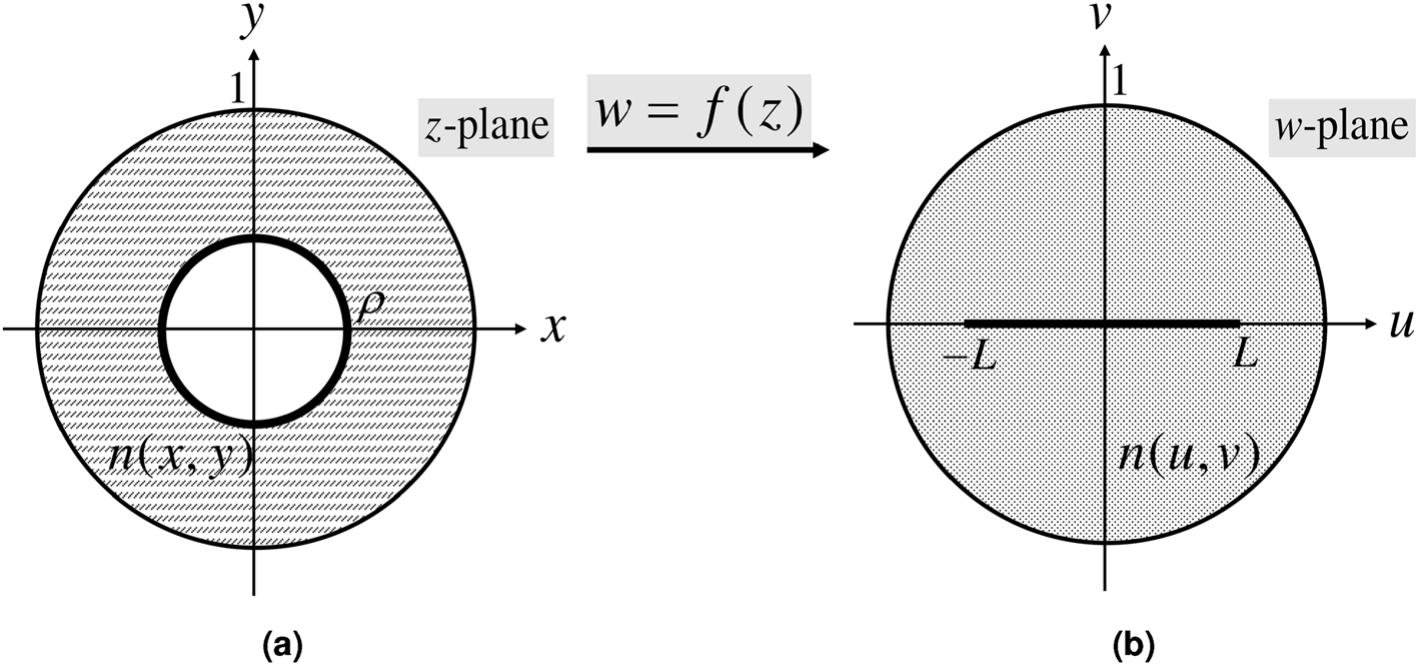
Schematics of the (**a**) physical space, and (**b**) virtual space for the directivity enhancement and cloaking of a cylindrical wire antenna.

Figure 1 depicts the schematics of virtual and physical spaces. An annulus with the inner radius of $\rho$ and the outer radius of one in the physical space is mapped to the doubly connected circular virtual space furnished with a slit $- L \le w \le L$ along the *u*-axis.

For the case of 2D simply connected shapes, Riemann’s mapping theorem implies that one can find a conformal mapping between two desired non-empty shapes. This theorem has been the critical factor for all transformation-optical researchers that employ conformal mappings. However, for the conformal mapping of 2D doubly connected regions, there exists another exciting theorem. After adapting the theorem to our case, it reads: “let *D* be a non-degenerate doubly connected region, then there exists a unique real number $\rho$, $0< \rho < 1$, such that there exists a one-to-one analytic function *f*(*z*) that maps the annulus $R_u$: $\rho< \left| z \right| < 1$ onto *D*. If the outer boundaries correspond to each other, then *f*(*z*) is determined up to a rotation of the annulus”. The unique value ${\rho ^{ - 1}}$ is referred to as the conformal modulus of *D*^66^. This means that for a given doubly connected virtual space, there exists a unique annulus with a specified inner radius of $\rho$ and an outer radius of 1.

There are four critical points to note regarding the configuration in Fig. 1:

1. The continuity of the transformation at the outer rim is sufficient to achieve the reflectionless property at the outer contour^67^. Hence, the outer boundary of both physical and virtual spaces is chosen alike. In the “Discussion” section, this point is discussed in greater detail.
2. There are two main options to improve the directivity of a cylindrical wire. Either one should map the outer contour of the virtual space to a square or a rectangle in the physical space, which is in contrast with the invisibility requirement, or one can transform the inner cylindrical wire in the physical space into a slit in the virtual space. An observer from the outside will perceive the electrical current placed on the inner cylinder of physical space as an electrical current excited on a slit. As a result, the directivity is enhanced since an electrical current excited on a slit radiates mainly along the direction normal to the slit, leading to two directive beams.
3. Based on the first and second points, the outside observer looking along the *x*-axis sees the perfect electric conductor (PEC) cylinder as the PEC slit. If the wave’s polarization is TM (magnetic field along the *z*-axis), the slit (or wire) will not disturb the incident TM wave and will eventually become invisible. Hence unidirectional cloaking can be achieved for the TM polarization. In the case of TE polarization (electric field along the *z*-axis), a perfect magnetic conductor (PMC) cylinder achieves invisibility^55^.
4. The shell is designed to increase the wire’s directivity, which emits TE polarized waves when excited by an electrical current. The dielectric medium designed by conformal transformation for this polarization is entirely efficient. However, for cloaking, TM polarization is involved. Conformal mapping results in a fully magnetic medium for this polarization, and obtaining a wideband magnetic response is difficult^65^. Although, if the structure’s size is electrically large, as it is in the geometrical optics (GO) regime, the dielectric medium designed based on the TE assumption works well for TM^68^.

It is worth noting that the propagation direction that the cylindrical wire is invisible for (along the *x*-axis) is perpendicular to the direction of the directivity enhancement (along the $\pm y$-axis) in our configuration.

A direct calculation of the mapping depicted in Fig. 1 is not possible. The mapping should be calculated by splitting the problem into a collection of conformal transformations. In this process, some preliminary conformal maps should be introduced. The fundamental analytic functions employed in the following calculations are available in the conformal mapping handbooks^69,70^.

**Figure 2:**
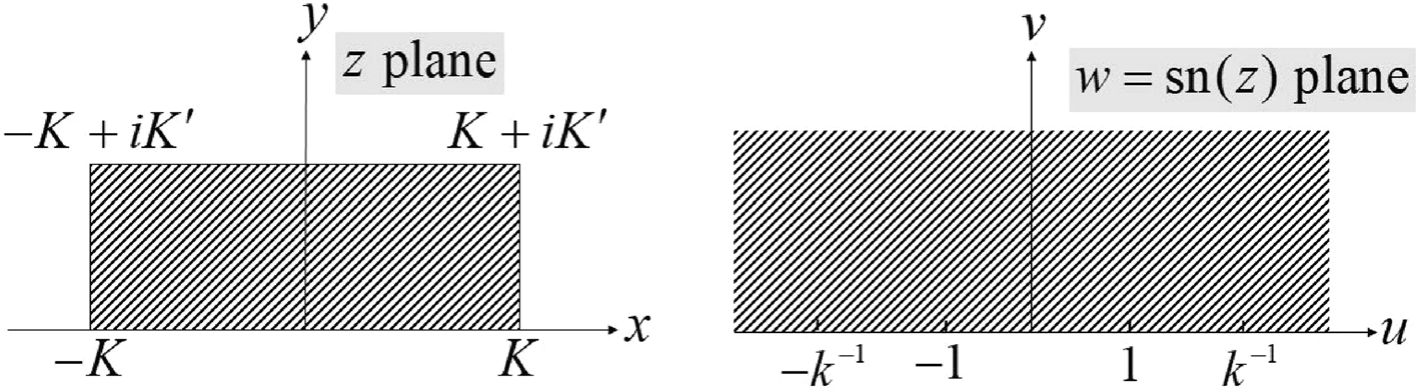
Schematic of the conformal mapping from a rectangle in the *z*-plane to the upper half of the *w*-plane^41^.

The conformal map from a rectangle to the upper half-space is introduced first. This mapping will be used later in the process. Figure 2 depicts this case.

Vertices $z = \pm K,\mathrm{{ }} \pm K + iK'$ are mapped to the vertices $w = \pm 1,\mathrm{{ }} \pm {k^{ - 1}}(0< k < 1)$. Table 1 summarizes the associated vertices.

**Table 1:** Vertices of the rectangle in the *z*-plane and their corresponding vertices in the upper half of the *w*-plane in Fig. 2.

*z*	*K*	$K + iK'$	$iK'$	$- K + iK'$	$- K$	0
$w = \mathrm{{sn}}(z,k)$	1	${k^{ - 1}}$	$\infty$	$- {k^{ - 1}}$	$-1$	0

The analytic function $w = \mathrm{{sn}}(z,k)$ known as the elliptic sine function provides such a transformation, where *k* is the modulus of the elliptical integrals. *K*(*k*) and $K'(k)$ are the complete and complementary complete elliptical integrals of the first with the $K'(k) = K(\sqrt{1 - {k^2}})$ property.

It is worth mentioning that if the modulus *k* is known, then the aspect ratio of the rectangle $K(k)/K'(k)$ is uniquely determined, and vice versa. It is helpful to define the intermediate parameter $q = {e^{ - \pi K'(k)/K(k)}}$, which depends on the aspect ratio and modulus *k*. Modulus *k* can be expressed as an infinite series of *q* following the below formula:

2 $$\begin{aligned} k(q) = 4\sqrt{q} \prod \limits _{n = 1}^\infty {{{\left( {\frac{{1 + {q^{2n}}}}{{1 + {q^{2n - 1}}}}} \right) }^4}}. \end{aligned}$$

The problem here is finding the analytic function $w=f(z)$ that maps the interior of the physical space’s annulus $\rho< \left| z \right| < 1$ to the interior of the virtual space’s unity circle $|w| = 1$, furnished with the slit $- L \le w \le L$. Applying the symmetry principle to the unity circle $\left| z \right| = 1$, we conclude that the analytic function $w = f(z)$ also maps the annulus $\rho< \left| z \right| < {\rho ^{ - 1}}$ to the entire *w*-plane, including the $- \infty \le w \le - {L^{ - 1}}, - L \le w \le L$, and ${L^{ - 1}} \le w \le \infty$ slits. Both domains have symmetry with respect to the real axis. Hence, if a mapping is found that can map the upper half of the annulus $\rho< \left| z \right| < {\rho ^{ - 1}}$ to the upper half of the *w*-plane, it is identical with $w = f(z)$ based on the symmetry principle, provided that it maps the points according to Table 2.

**Table 2:** Vertices correspondence for the conformal map of the upper half of the annulus $\rho< \left| z \right| < {\rho ^{ - 1}}$ to the upper half of the *w*-plane including the $- \infty \le w \le - {L^{ - 1}}, - L \le w \le L$, and ${L^{ - 1}} \le w \le \infty$ slits.

*z*	$\rho$	$-\rho$	${\rho ^{ - 1}}$	${\rho ^{ - 1}}$
*w*	*L*	$-L$	$L^{ - 1}$	$-L^{ - 1}$

**Figure 3:**
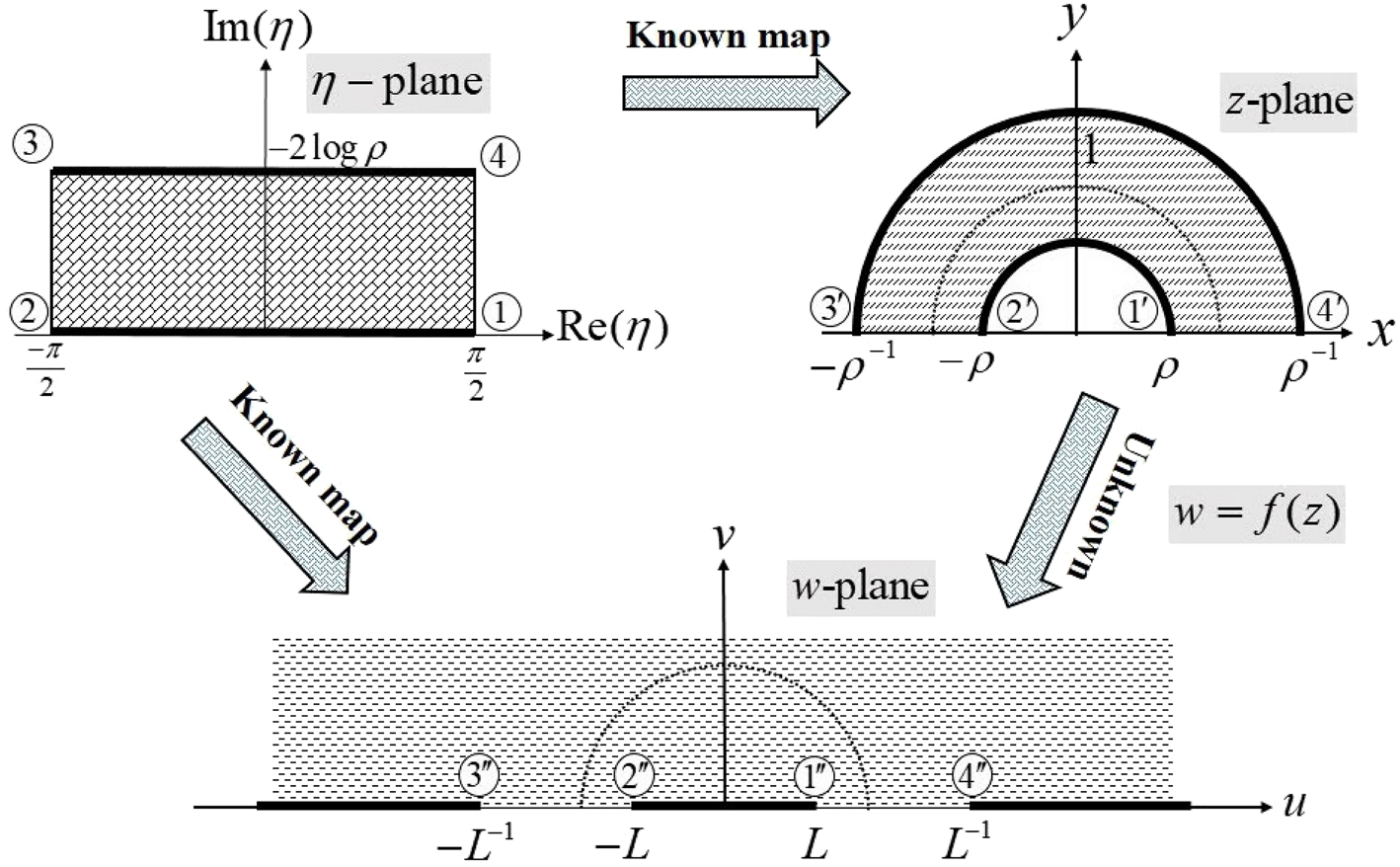
Schematic of the procedure for deriving the conformal map $w=f(z)$.

The procedure for finding the required conformal map is depicted in Fig. 3, where the unknown mapping is derived indirectly using two known conformal maps.

The problem, by far, includes finding a transformation that maps the upper half of the annulus $\rho< \left| z \right| < {\rho ^{ - 1}}$ to the upper half of the *w*-plane. On the other hand, the transformation from a rectangle to the upper half of the *w*-plane has already been derived (see Fig. 2). Hence, finding a conformal map between the rectangle and the upper half of the annulus $\rho< \left| z \right| < {\rho ^{ - 1}}$ is the key to finding the solution.

The exponential function $z = i\rho {e^{ - i\eta }}$ maps the rectangular region $- \pi /2< \mathrm{{Re}}\{ \eta \} < \pi /2,$ $0< \mathrm{{Im}}\{ \eta \} < - 2\log \rho$ onto the upper half of the annulus $\rho< \left| z \right| < {\rho ^{ - 1}}$. Table 3 summarizes the points involved in the mapping.

**Table 3:** Vertices of the rectangle in the $\eta$-plane and their corresponding vertices in the upper half of the annulus $\rho< \left| z \right| < {\rho ^{ - 1}}$.

$\eta$	$\frac{\pi }{2}$	$-\frac{\pi }{2}$	$\frac{\pi }{2} - 2i\log \rho$	$-\frac{\pi }{2} - 2i\log \rho$
$z = i\rho {e^{ - i\eta }}$	$\rho$	$-\rho$	${\rho ^{ - 1}}$	$-{\rho ^{ - 1}}$

The vertices and their mapped counterparts in Fig. 3 are labeled with numbers according to Tables 2 and 3.

Based on Fig. 3, the analytic function $w = f(i\rho {e^{ - i\eta }})$ maps the rectangle in the $\eta$-plane to the upper half of the *w*-plane. Based on Tables 2 and 3, the correspondence in Table 4 is established.

**Table 4:** The established correspondence based on Tables 2 and 3.

$\eta$	$\frac{\pi }{2}$	$-\frac{\pi }{2}$	$\frac{\pi }{2} - 2i\log \rho$	$-\frac{\pi }{2} - 2i\log \rho$
$L^{ - 1}f(i\rho {e^{ - i\eta }})$	1	$-1$	$L^{ - 2}$	$-L^{ - 2}$

Comparing Tables 1 and 4, leads to the following equalities:

3 $$\begin{aligned} \left\{ \begin{array}{l} {L^{ - 1}}f(i\rho {e^{ - i\eta }}) = \mathrm{{sn}}\frac{{2K}}{\pi }\eta , \\ \frac{{K'}}{K} = - \frac{4}{\pi }\log \rho , \\ k = {L^2}. \end{array} \right. \end{aligned}$$

In conclusion, the following analytic function $w=f(z)$ maps the interior of the physical space’s annulus $\rho< \left| z \right| < 1$ to the interior of the virtual space’s unity circle $|w| = 1$ furnished with the slit $- L \le w \le L$:

4 $$\begin{aligned} w = f(z) = \sqrt{k} \mathrm{{sn}}\left( {\frac{{2iK}}{\pi }\log \frac{z}{\rho } + K;k} \right) ;k = k({\rho ^4}), \end{aligned}$$

where the *q* parameter associated with the above conformal mapping is $q = {e^{ - \pi K'(k)/K(k)}}=\rho ^{4}$ based on Eq. (3). According to Eq. (2) and the $k=L^{2}$ relation, the following formula relates the half-length of the virtual space’s slit *L* to the inner radius of the physical space’s annulus $\rho$:

5 $$\begin{aligned} L(\rho ) = 2\rho \prod \limits _{n = 1}^\infty {{{\left( {\frac{{1 + {\rho ^{8n}}}}{{1 + {\rho ^{8n - 4}}}}} \right) }^2}}. \end{aligned}$$

Following Eq. (1), the refractive index of the physical space is derived:

6 $$\begin{aligned} n(x,y) =\frac{{2iK\sqrt{k} }}{{\pi z}}\mathrm{{cn}}\left( {\frac{{2iK}}{\pi }\log \frac{z}{\rho } + K;k} \right) \mathrm{{dn}}\left( {\frac{{2iK}}{\pi }\log \frac{z}{\rho } + K;k} \right) ;k = k({\rho ^4}), \end{aligned}$$

where cn and dn are the elliptic cosine and the delta amplitude functions, respectively.

The inverse of the mapping in Eq. (4) can be derived using the definition of the $\mathrm{{s}}{\mathrm{{n}}^{ - 1}}$ function.

7 $$\begin{aligned}z = {f^{ - 1}}(w) = \rho {e^{\frac{\pi }{{2iK}}\left( {\int _0^{\frac{w}{{\sqrt{k} }}} {\frac{{dt}}{{\sqrt{(1 - {t^2})(1 - {k^2}{t^2})} }} - K} } \right) }};k = k({\rho ^4}). \end{aligned}$$

The above integral can be solved numerically or by using “InverseJacobiSN” in Mathematica^71^.

**Figure 4:**
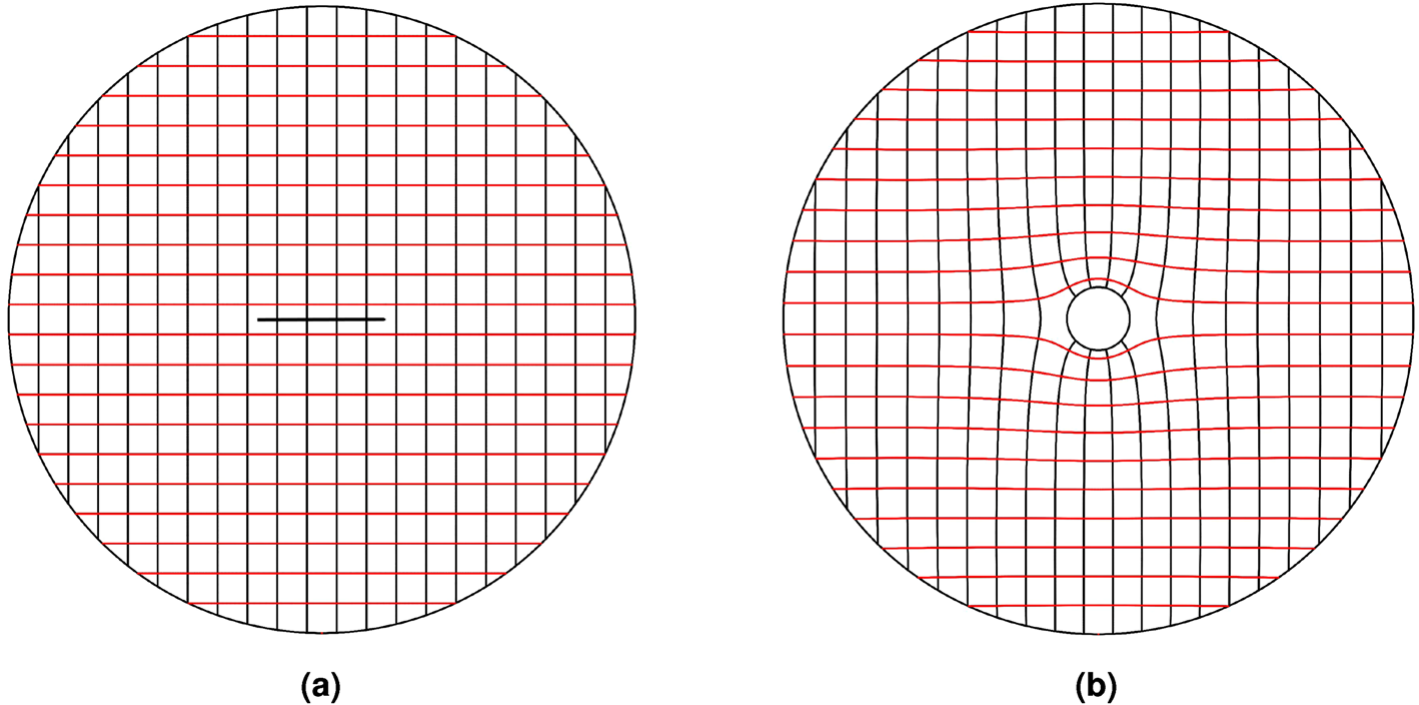
Grid transformation between the virtual and physical spaces. (**a**) Cartesian grids in the virtual space, and (**b**) the corresponding mapped ones in the physical space.

**Figure 5:**
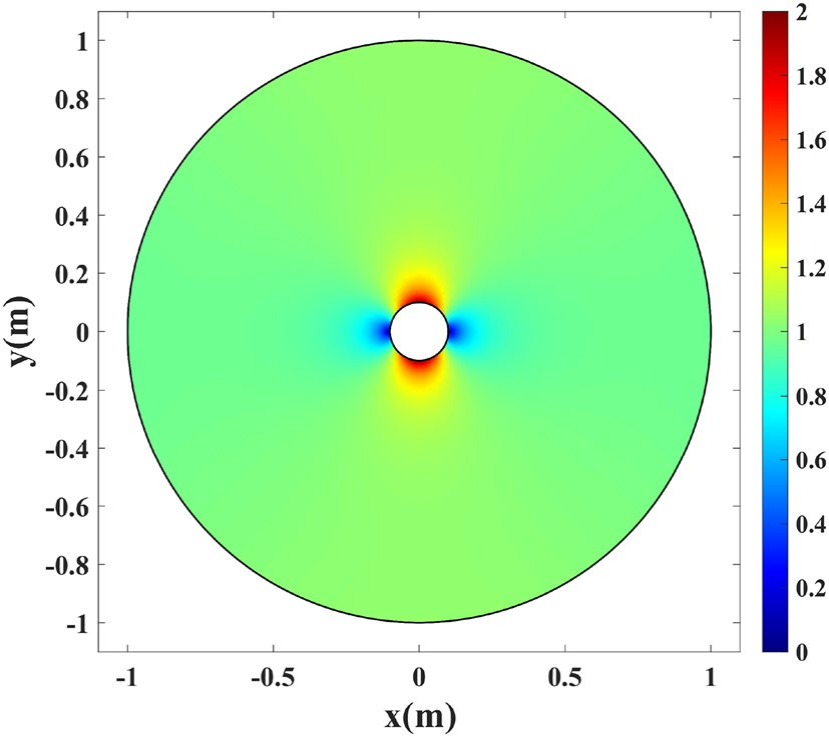
The refractive index profile of the physical space in Fig. 1a with the inner radius of $\rho =0.1$ m.

Here, the radius of the wire antenna equals $\rho =0.1$ m. The corresponding slit length of $2L=0.4$ m is calculated following Eq. (5). Figure 4 depicts the Cartesian grids inside the virtual space and their mapped counterparts in the physical space. The *v*-constant grids representing a set of rays propagating along the *u*-axis in the virtual space are guided around the wire as they are mapped to the physical space. Hence, the unidirectional cloaking effect is to be expected. Furthermore, the mapped *u*-constant grids near the wire are perpendicular to the circumference of the wire. As a result, it is expected that rays emanating from the wire perpendicular to its boundary will be gradually focused along the $\pm y$ direction by the transformation medium, resulting in two directive beams. Following Eq. (6), the refractive index is calculated and illustrated in Fig. 5.

## Simulation results

**Figure 6:**
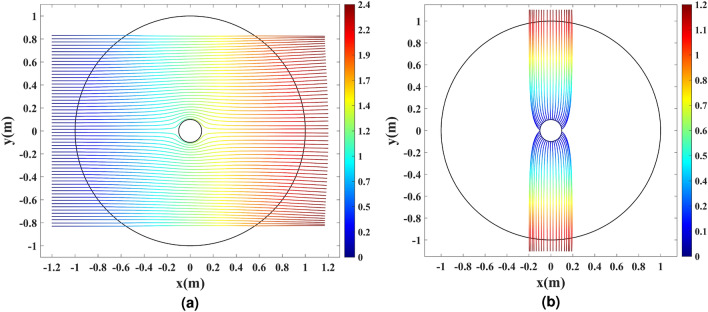
Ray-tracing simulation of the physical space with the refractive index depicted in Fig. 5. (**a**) The cloaking effect for 60 parallel rays launched along the *x*-axis. (**b**) The directivity enhancement effect for 38 equally-spaced rays launched from the inner rim. The color expression presents the optical path length in meters.

Ray-tracing and full-wave simulations are carried out to verify the of the device’s functionality. The ray-tracing simulations assess the response of the device in the GO regime. First, 60 parallel rays are launched along the *x*-axis from the left side of the simulation domain to evaluate the cloaking functionality. The results are depicted in Fig. 6a, where the color expression represents the optical path length in meters. It is seen that the rays are guided around the wire, implying that the cylindrical wire is unidirectionally invisible. To verify the directivity enhancement, 38 equally-spaced rays are generated in the direction perpendicular to the boundary of the wire. The results are illustrated in Fig. 6b, where the rays generated from the wire’s surface are gathered along the $\pm y$ direction by the dielectric shell. This confirms the directivity improvement as the omnidirectional pattern of the cylindrical wire changes to a pattern with two beams along the $\pm y$ direction.

**Figure 7:**
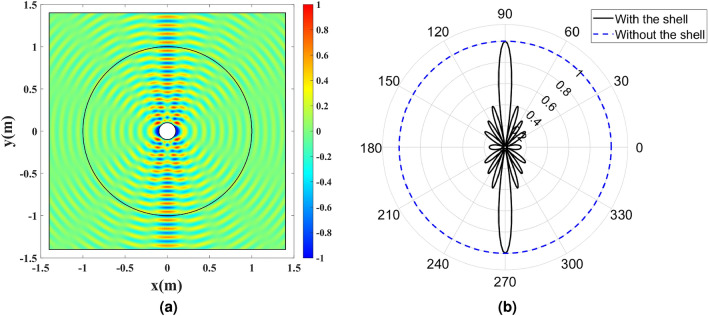
The cylindrical wire enclosed by the refractive index profile depicted in Fig. 5 and excited by the uniform electric current density ${J_z}$ at 3 GHz. (**a**) The amplitude of ${E_z}$, and (**b**) the normalized far-field pattern with and without the dielectric shell.

The full-wave simulations are carried out at the frequency of 3 GHz. The directivity enhancement is investigated by exciting the cylindrical wire with an out-of-plane electrical current density ${J_z}$. Such a current produces TE waves. The amplitude of the electric field ${E_z}$ and the far-field pattern with and without the dielectric shell are depicted in Fig. 7. The directivity enhancement is seen by comparing the results with the omnidirectionally radiating bare wire.

**Figure 8:**
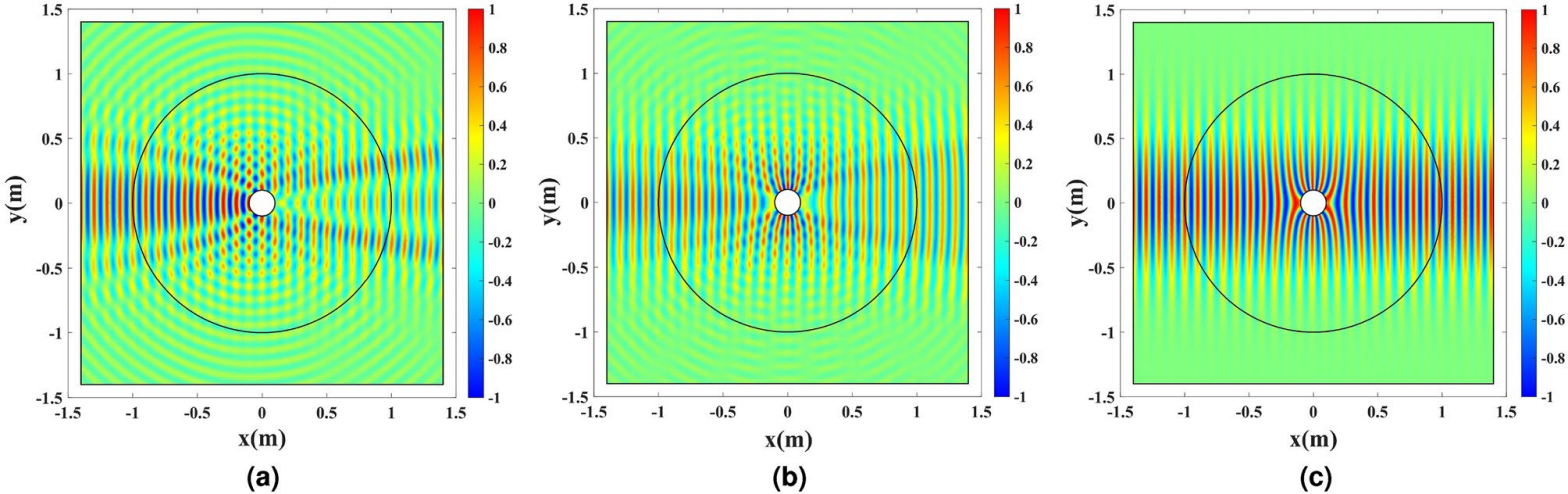
The amplitude of ${H_z}$ for a TM Gaussian wave illuminating the wire antenna covered with (**a**) no cloak, (**b**) a pure dielectric cloak, and (**c**) a pure magnetic cloak.

To investigate the cloaking functionality, a TM Gaussian wave that propagates along the *x*-axis illuminates the structure from the left boundary. The magnetic field amplitude ${H_z}$ is depicted in Fig. 8 for three cases: the bare wire, and the cases where the refractive index is realized by pure permittivity and permeability. As mentioned in “Introduction”, the pure permittivity performs similarly to the pure permeability if the structure is electrically large. However, perfect cloaking is obtained for the case where pure permeability is employed. The pure permeability case is simulated only for comparison’s sake.

**Figure 9:**
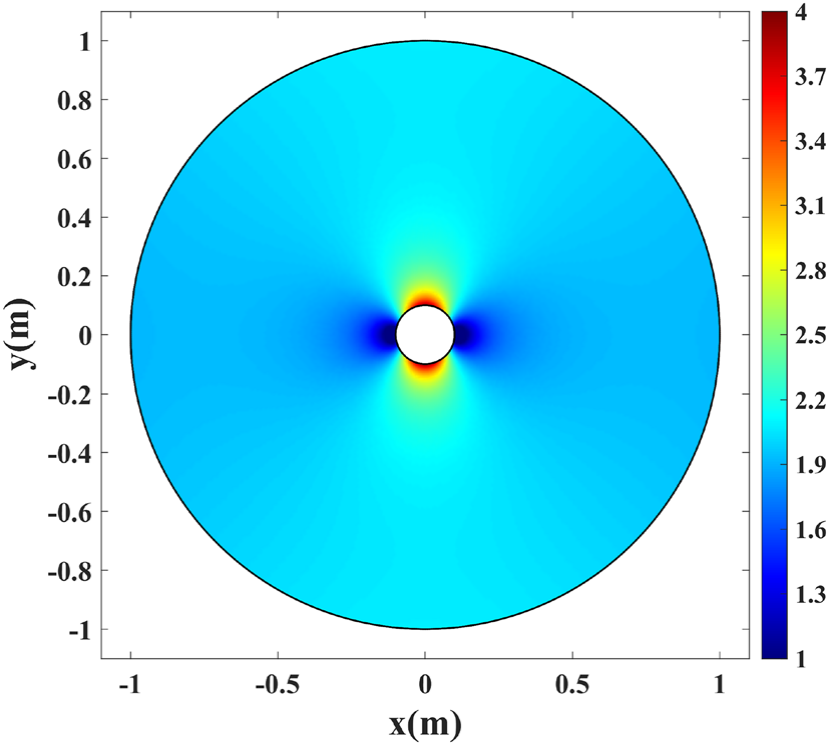
The modified refractive index profile in Fig. 5. The background refractive index is changed to 2 and the superluminal index values are replaced with a unity refractive index.

To bring the refractive index profile in Fig. 5 one step closer to realization, we have changed the background refractive index to 2 and replaced the below unity values with one. Figure 9 depicts the modified refractive index.

**Figure 10:**
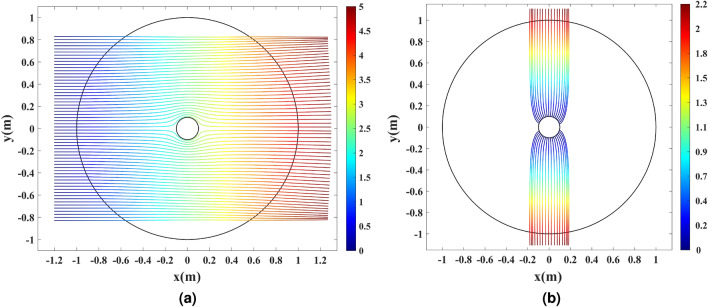
Ray-tracing simulation of the physical space with the refractive index depicted in Fig. 9. (**a**) The cloaking effect for 60 parallel rays launched along the *x*-axis. (**b**) The directivity enhancement effect for 36 equally-spaced rays launched from the inner rim. The color expression presents the optical path length in meters.

Figure 10 illustrates the ray-tracing results for the refractive index profile shown in Fig. 9, where the color expression represents the optical path length.

**Figure 11:**
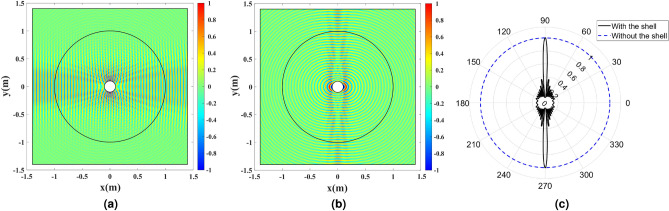
Full-wave simulation results for the cylindrical wire enclosed by the refractive index depicted in Fig. 9. (**a**) The amplitude of ${H_z}$ for a TM Gaussian wave illuminating the structure. (**b**) The amplitude of ${E_z}$ for the wire excited by the uniform electric current density ${J_z}$ at 3 GHz. (**c**) The normalized far-field pattern with and without the dielectric shell.

The full-wave simulation results are depicted in Fig. 11. To illuminate the structure, a TM Gaussian wave propagating along the *x*-axis is excited from the left boundary. The magnetic field amplitude ${H_z}$ is depicted in Fig. 11a. Also, the directivity enhancement functionality is shown by exciting the cylindrical wire with an electrical current density ${J_z}$. The amplitude of the electric field ${E_z}$ and the far-field pattern with and without the dielectric shell are depicted in Fig. 11b,c.

The ray-tracing and full-wave simulation results for the dielectric shell in Fig. 9 show that the device maintains its functionality after manipulating the refractive index.

**Figure 12:**
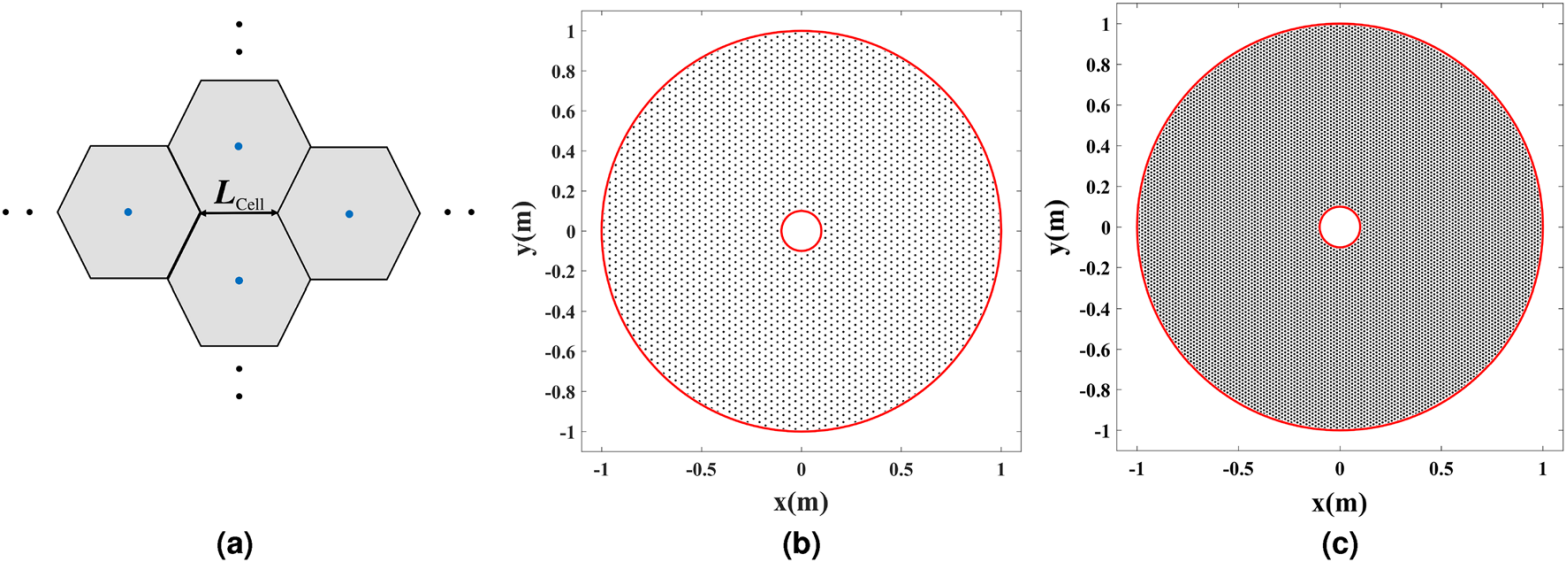
Discretization of refractive index profile in Fig. 9. (**a**) The hexagonal lattice configuration. (**b**) Sampling points for $L_\mathrm{{Cell}}=\lambda /5$. (**c**) Sampling points for $L_\mathrm{{Cell}}=\lambda /10$.

Next, the discretized refractive index is examined. A hexagonal lattice is selected to effectively tessellate the refractive index profile. Figure 12a depicts the arrangement of the hexagonal cells with the side length of $L_\mathrm{{Cell}}$. Two cases are investigated where the side length of the hexagons $L_\mathrm{{Cell}}$ equals $\lambda /5$ and $\lambda /10$ for the frequency of 10 GHz. The sampling points (centers of the hexagon cells) are illustrated in Figs. 12b,c. Based on the refractive index profile and the local wavelength, both samplings are coarse near the cylindrical wire.

**Figure 13:**
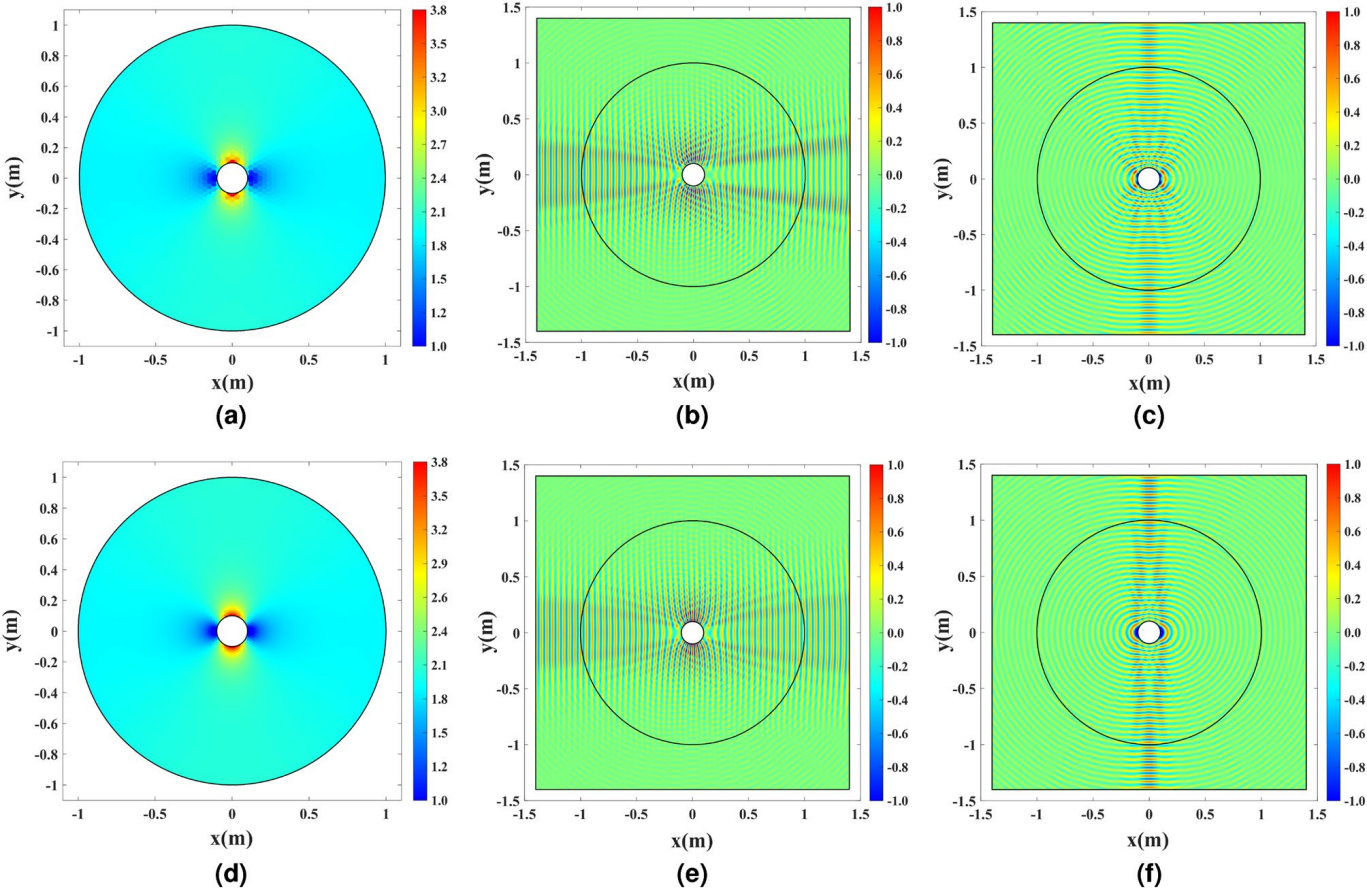
Full-wave simulation results for the cylindrical wire enclosed by the discretized refractive index. (**a**) Discretized refractive index profile, (**b**) the amplitude of ${H_z}$ for a TM Gaussian wave illuminating the structure, and (**c**) the amplitude of ${E_z}$ for the wire excited by the uniform electric current density ${J_z}$ at 3 GHz for $L_\mathrm{{Cell}}=\lambda /5$. (**d**) Discretized refractive index profile, (**e**) the amplitude of ${H_z}$ for a TM Gaussian wave illuminating the structure, and (**f**) the amplitude of ${E_z}$ for the wire excited by the uniform electric current density ${J_z}$ at 3 GHz for $L_\mathrm{{Cell}}=\lambda /10$.

Full-wave simulations are carried out to compare the results of the sampled shell depicted in Figs. 12b,c, with the ones illustrated in Fig. 11a,b. Results are presented in Fig. 13.

It is seen that the cloaking and the directivity enhancing properties degrade for the $L_\mathrm{{Cell}}=\lambda /5$ case since the sampling is very coarse. However, the results obtained for the $L_\mathrm{{Cell}}=\lambda /10$ case are almost identical to the Fig. 11a,b results where the refractive index is continuous.

## Discussion

It is worth investigating the perfectness of the invisibility provided by the dielectric shell. The proposed conformal map transforms the circular outer boundaries of the physical space and the virtual space to each other. This aspect contributes to maintaining the map’s continuity at the outer boundary and, as a result, avoiding reflections. However, this effect is inherently not perfect. Based on the uniqueness theorem for analytic functions, if the values of two conformal maps on a specific boundary (outer rims of both spaces) equal each other, the two conformal maps are equal (Theorem 10.39)^72^. It means that the perfect transformation continuity requirement for cloaking $(x' = x,\mathrm{{ }}y' = y,\mathrm{{ }}z' = z)$, forces the conformal map to be a unity transformation, which is not intended. In fact, if the radial and angular coordinates of the virtual and physical spaces are denoted as $(R,\psi )$ and $(r,\phi )$, the conformal mapping provided in Eq. (4) only ensures the perfect equality of the radial components at the outer contour $(R=r)$. Due to the uniqueness theorem, the angular components can not be equal simultaneously $(\psi \ne \phi )$. As the radius of the wire increases, the angular components deviate more.

**Figure 14:**
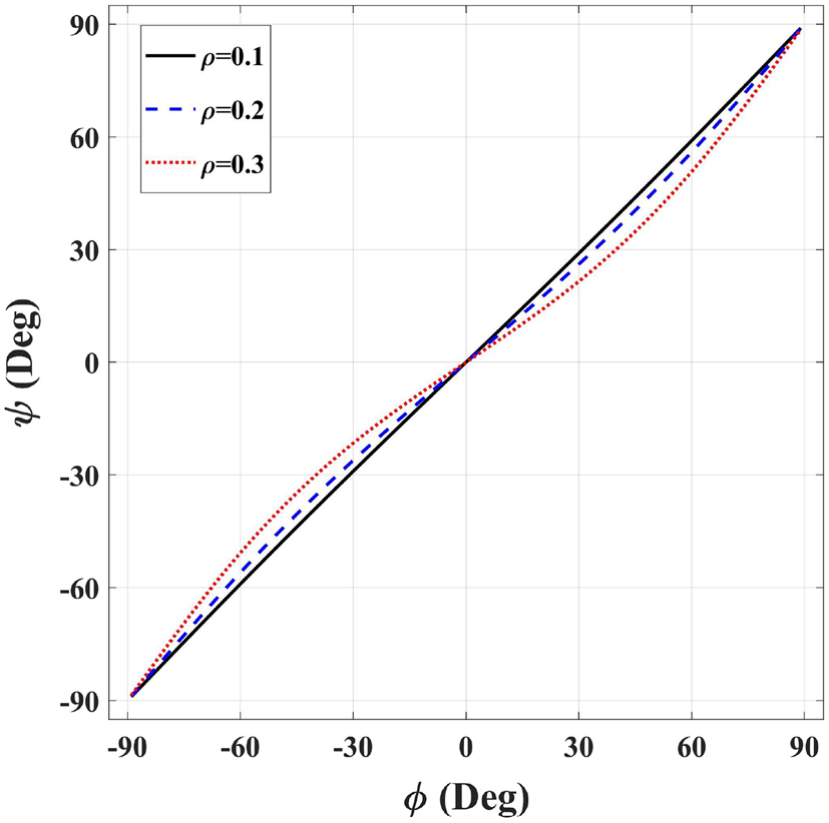
The angular coordinate of the virtual space $\psi$ versus the angular coordinate of the physical space $\phi$ on the physical space’s outer contour for $\rho = 0.1,\mathrm{{ }}0.2$, and 0.3 m.

Figure 14 plots $\psi$ versus $\phi$ for the inner radius values of $\rho = 0.1, 0.2$, and 0.3 m. It is seen that the angular coordinate equality between the two spaces is quite good for a small inner radius value. This equality becomes increasingly absolute as $\rho$ converges to zero, making the physical and virtual spaces similar, representing a unity transformation.

**Figure 15:**
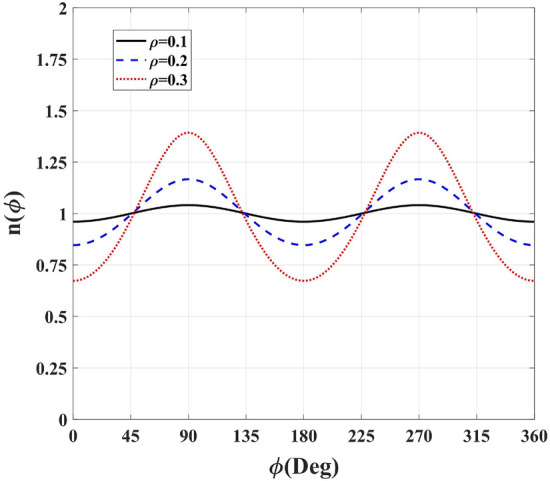
The refractive index of the physical space’s outer contour versus $\phi$ for $\rho = 0.1,\mathrm{{ }}0.2$, and 0.3 m.

This perfectness of the cloaking can be studied from a different point of view. A refractive index of one $(\left| {f'(z)} \right| =1)$ on the outer boundary is sufficient for avoiding reflections. Here, the complex variables $z = x + iy = r{e^{i\varphi }}$ and $w = f(r{e^{i\varphi }}) = u + iv = R{e^{i\psi }}$ describe the physical and virtual spaces, respectively. Following Eq. (1), the refractive index formula in the polar coordinates follows the below relation:

8 $$\begin{aligned} n = \left| {f'(z)} \right| = \sqrt{R_r^2 + {{(R{\psi _r})}^2}} = \frac{{\sqrt{R_\varphi ^2 + {{(R{\psi _\varphi })}^2}} }}{r}, \end{aligned}$$

where the subscripts denote partial derivatives and the Cauchy–Riemann conditions in polar coordinates ${u_r} = {v_\varphi }/r,\mathrm{{ }}{v_r} = - {u_\varphi }/r$ are considered. The proposed conformal mapping ensures $R=r$ equality at the outer rim. Hence, the refractive index at the exterior border of the cloak $(R=r=1)$ equals $\sqrt{R_r^2 + \psi _r^2}$ or $\sqrt{R_\varphi ^2 + \psi _\varphi ^2}$ equivalently. Figure 15 plots the refractive index of the physical space’s outer contour for the inner radius values of $\rho = 0.1, 0.2$, and 0.3 m. It is seen that the refractive index begins to deviate from unity as the inner radius increases.

**Figure 16:**
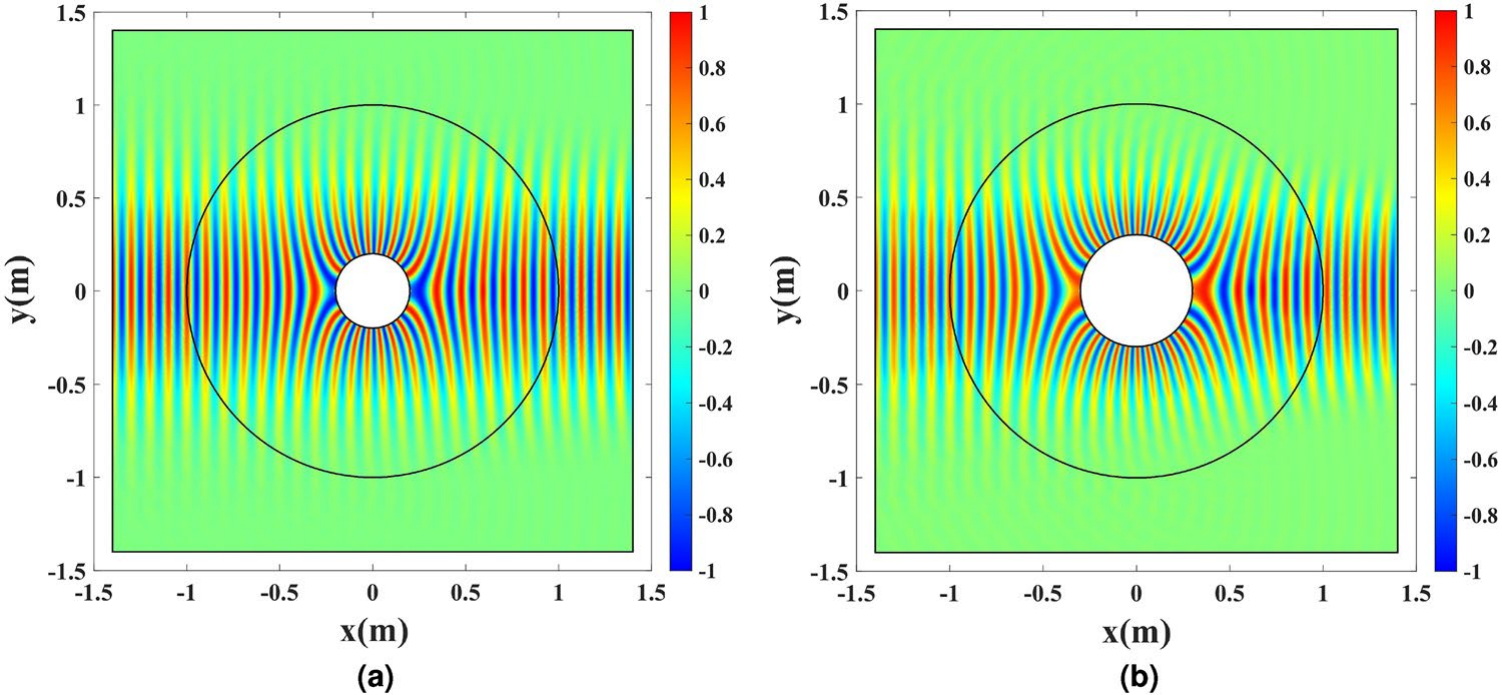
A TM Gaussian wave illuminates the wire antenna enclosed by a pure magnetic cloak. (**a**) $\rho =0.2$ m, and (**b**) $\rho =0.3$ m.

To verify the perfectness of the unidirectional cloaking, the structure from the simulation section is illuminated by a TM Gaussian wave that propagates along the *x*-axis. The refractive index is realized by a pure permeability to eliminate any imperfection from using a pure dielectric material for the TM wave. Figure 16 shows the results for the cases in which the inner radius values of $\rho =0.2$, and 0.3 m are chosen, resulting in excellent cloaking. The case with the inner radius of $\rho =0.1$ m has already been depicted in Fig. 8c.

It’s also worth looking into the Zhukovsky mapping^56^. The Zhukovsky transformation $w = f(z) = z + {\rho ^2}/z$ maps the inner wire with the radius of $\rho$ in the physical space to a slit with the half-length of $L = 2\rho$ in the virtual space. In addition, the outer contour of physical space $|z| = 1$ is mapped to an ellipse with major and minor axes equal to $1 + {\rho ^2}$ and $1 - {\rho ^2}$, respectively. As $\rho$ increases, the physical space’s circular outer boundary transforms into an ellipse in the virtual space with a larger axial ratio. Hence, the outer boundaries of physical and virtual spaces become less similar.

Comparing the slit half-length for the Zhukovsky case $L = 2\rho$ with Eq. (5), it is clear that Eq. (5) contains an additional product series term. The value of the series decreases as $\rho$ increases, and it approaches one as $\rho$ approaches zero. In conclusion, for the mapping problem depicted in Fig. 1, the conformal mapping of Eq. (4) converges to the Zhukovsky mapping for very small values of $\rho$.

As the theory implies and as seen in the cases of the Zhukovsky mapping and the conformal mapping presented in Eq. (4), for a specific doubly connected physical space (annulus shape in this work), there exists a unique value for the slit length embedded in the virtual space. The value of slit length is directly related to the conformal module of the physical space ${\rho ^{ - 1}}$. The numerical method presented in the literature employs Dirichlet and Neumann boundary conditions on the physical space’s inner and outer boundaries to ensure the continuity of the transformation on the outer rim and the orthogonality of the meshes^65^. However, the boundary conditions cannot establish any correspondence between ${\rho ^{ - 1}}$ and the slit length. The method presented in the literature offers no solution for conformal module matching. As a result, as the radius of the wire increases, the numerically calculated transformation deviates substantially from being conformal.

## Conclusion

The doubly connected annulus in the physical space is mapped to a circle furnished with a slit using a closed-form, strictly conformal map. Such a mapping provides unidirectional cloaking while improving the directivity of a cylindrical wire antenna. The conformal mapping leads to an isotropic dielectric material. Both ray-tracing and full-wave simulations confirm the proposed design method.

## Data Availability

The datasets used and/or analysed during the current study available from the corresponding author on reasonable request.

## References

[1] Leonhardt U (2006). Optical conformal mapping. Science.

[2] Pendry JB (2006). Controlling electromagnetic fields. Science.

[3] Chen H, Chan CT (2007). Transformation media that rotate electromagnetic fields. Appl. Phys. Lett..

[4] Kwon D-H, Werner DH (2008). Polarization splitter and polarization rotator designs based on transformation optics. Opt. Express.

[5] Chen H (2009). Design and experimental realization of a broadband transformation media field rotator at microwave frequencies. Phys. Rev. Lett..

[6] Rahm M (2008). Design of electromagnetic cloaks and concentrators using form-invariant coordinate transformations of maxwell’s equations. Photonics Nanostruct. Fundam. Appl..

[7] Sadeghi MM, Xu L, Nadgaran H, Chen H (2015). Optical concentrators with simple layered designs. Sci. Rep..

[8] Li J, Pendry JB (2008). Hiding under the carpet: A new strategy for cloaking. Phys. Rev. Lett..

[9] Liu R (2009). Broadband ground-plane cloak. Science.

[10] Ma HF, Cui TJ (2010). Three-dimensional broadband ground-plane cloak made of metamaterials. Nat. Commun..

[11] Zhang B, Chan T, Wu B-I (2010). Lateral shift makes a ground-plane cloak detectable. Phys. Rev. Lett..

[12] Chen X (2011). Macroscopic invisibility cloaking of visible light. Nat. Commun..

[13] Kadic M (2012). Transformation plasmonics. Nanophotonics.

[14] Liu Y, Zentgraf T, Bartal G, Zhang X (2010). Transformational plasmon optics. Nano Lett..

[15] Wang S-Y, Liu S-B, Guo Y-N, Ghen C (2013). A v-shaped cavity camouflage coating. Opt. Laser Technol..

[16] Eskandari H, Tyc T (2019). Controlling refractive index of transformation-optics devices via optical path rescaling. Sci. Rep..

[17] Mousavi SSS, Majedi MS, Eskandari H (2017). Design and simulation of polarization transformers using transformation electromagnetics. Optik.

[18] Eskandari H, Majedi MS, Attari AR (2017). Design of reflectionless non-magnetic homogeneous polarization splitters with minimum anisotropy based on transformation electromagnetics. J. Opt. Soc. Am. B.

[19] Eskandari H, Majedi MS, Attari AR (2017). Non-reflecting non-magnetic homogeneous polarization splitter and polarization deflector design based on transformation electromagnetics. Optik.

[20] Eskandari H, Attari AR, Majedi MS (2018). Design of polarization splitting devices with ideal transmission and anisotropy considerations. J. Opt. Soc. Am. B.

[21] Rahm M, Roberts DA, Pendry JB, Smith DR (2008). Transformation-optical design of adaptive beam bends and beam expanders. Opt. Express.

[22] Emiroglu CD, Kwon D-H (2010). Impedance-matched three-dimensional beam expander and compressor designs via transformation optics. J. Appl. Phys..

[23] Schmiele M, Varma VS, Rockstuhl C, Lederer F (2010). Designing optical elements from isotropic materials by using transformation optics. Phys. Rev. A.

[24] Yao K, Jiang X (2011). Designing feasible optical devices via conformal mapping. J. Opt. Soc. Am. B.

[25] Wu Q (2013). Transformation optics inspired multibeam lens antennas for broadband directive radiation. IEEE Trans. Antennas Propag..

[26] Ebrahimpouri M, Quevedo-Teruel O (2017). Bespoke lenses based on quasi-conformal transformation optics technique. IEEE Trans. Antennas Propag..

[27] Aghanejad I, Abiri H, Yahaghi A (2012). Design of high-gain lens antenna by gradient-index metamaterials using transformation optics. IEEE Trans. Antennas Propag..

[28] Aghanejad I, Abiri H, Yahaghi A (2016). High-gain planar lens antennas based on transformation optics and substrate-integrated waveguide (SIW) technology. Progress Electromagn. Res. C.

[29] Eskandari H, Saviz S, Tyc T (2021). Directivity enhancement of a cylindrical wire antenna by a graded index dielectric shell designed using strictly conformal transformation optics. Sci. Rep..

[30] Xu HY, Zhang B, Barbastathis G, Sun HD (2011). Compact optical waveguide coupler using homogeneous uniaxial medium. J. Opt. Soc. Am. B.

[31] Huang L (2013). A general transformation for compact waveguide coupler by using homogeneous media. Photon. Nanostruct. Fundam. Appl..

[32] Chen C (2013). A shifted waveguide connector combined with a photonic crystal filter designed by transformation optics. Opt. Laser Technol..

[33] Eskandari H, Majedi MS, Attari AR (2017). Reflectionless compact nonmagnetic optical waveguide coupler design based on transformation optics. Appl. Opt..

[34] García-Meca C (2011). Squeezing and expanding light without reflections via transformation optics. Opt. Express.

[35] Wu Q, Turpin JP, Werner DH (2012). Integrated photonic systems based on transformation optics enabled gradient index devices. Light Sci. Appl..

[36] Eskandari H, Attari AR, Majedi MS (2017). Reflectionless design of a nonmagnetic homogeneous optical waveguide coupler based on transformation optics. J. Opt. Soc. Am. B.

[37] Eskandari H, Quevedo-Teruel O, Attari AR, Majedi MS (2019). Transformation optics for perfect two-dimensional non-magnetic all-mode waveguide couplers. Opt. Mater. Express.

[38] Roberts DA, Kundtz N, Smith DR (2009). Optical lens compression via transformation optics. Opt. Express.

[39] Quevedo-Teruel O (2013). Transformation optics for antennas: Why limit the bandwidth with metamaterials?. Sci. Rep..

[40] Ebrahimpouri M, Quevedo-Teruel O (2019). Ultrawideband anisotropic glide-symmetric metasurfaces. IEEE Antennas Wirel. Propag. Lett..

[41] Eskandari H, Majedi MS, Attari AR, Quevedo-Teruel O (2019). Elliptical generalized Maxwell fish-eye lens using conformal mapping. New J. Phys..

[42] Taskhiri MM (2021). Axis-symmetric ellipsoidal lens antenna design with independent e and h radiation pattern beamwidth. Opt. Laser Technol..

[43] Kadera P (2022). Wide-angle ceramic retroreflective luneburg lens based on quasi-conformal transformation optics for mm-wave indoor localization. IEEE Access.

[44] Xu X (2011). Broad band invisibility cloak made of normal dielectric multilayer. Appl. Phys. Lett..

[45] Zhang J, Liu L, Luo Y, Zhang S, Mortensen NA (2011). Homogeneous optical cloak constructed with uniform layered structures. Opt. Express.

[46] Landy NI, Padilla WJ (2009). Guiding light with conformal transformations. Opt. Express.

[47] Xu L, Chen H (2014). Conformal transformation optics. Nat. Photon..

[48] Chang Z, Zhou X, Hu J, Hu G (2010). Design method for quasi-isotropic transformation materials based on inverse laplace’s equation with sliding boundaries. Opt. Express.

[49] Luo Y (2009). A rigorous analysis of plane-transformed invisibility cloaks. IEEE Trans. Antennas Propag..

[50] Xi S, Chen H, Wu B-I, Kong JA (2009). One-directional perfect cloak created with homogeneous material. IEEE Microw. Wirel. Compon. Lett..

[51] Landy N, Smith DR (2013). A full-parameter unidirectional metamaterial cloak for microwaves. Nat. Mater..

[52] Zheng B (2019). Experimental realization of an extreme-parameter omnidirectional cloak. Research.

[53] Jiang WX (2008). Invisibility cloak without singularity. Appl. Phys. Lett..

[54] Jiang WX, Feng Ma H, Cheng Q, Cui TJ (2010). A class of line-transformed cloaks with easily realizable constitutive parameters. J. Appl. Phys..

[55] Ma Y (2013). First experimental demonstration of an isotropic electromagnetic cloak with strict conformal mapping. Sci. Rep..

[56] Chen H, Leonhardt U, Tyc T (2011). Conformal cloak for waves. Phys. Rev. A.

[57] Xu L, Chen H, Tyc T, Xie Y, Cummer SA (2016). Perfect conformal invisible device with feasible refractive indexes. Phys. Rev. B.

[58] Xu L, Tyc T, Chen H (2019). Conformal optical devices based on geodesic lenses. Opt. Express.

[59] Liu Y, Sun F, He S (2019). Omnidirectional conformal cloak without geometrical dispersion. Phys. Rev. Appl..

[60] Wang Z, Liu Y, Cheng T, Sun F, He S (2020). Designing conformal cloaks by manipulating structures directly in the physical space. Opt. Express.

[61] Zhang K, Wang Y, Burokur SN, Wu Q (2021). Generating dual-polarized vortex beam by detour phase: From phase gradient metasurfaces to metagratings. IEEE Trans. Microw. Theory Tech..

[62] Wang Y (2022). Perfect control of diffraction patterns with phase-gradient metasurfaces. ACS Appl. Mater. Interfaces.

[63] Eskandari H, Albadalejo-Lijarcio JL, Zetterstrom O, Tyc T, Quevedo-Teruel O (2021). H-plane horn antenna with enhanced directivity using conformal transformation optics. Sci. Rep..

[64] Keivaan A, Fakheri MH, Abdolali A, Oraizi H (2017). Design of coating materials for cloaking and directivity enhancement of cylindrical antennas using transformation optics. IEEE Antennas Wirel. Propag. Lett..

[65] Fakheri MH, Abdolali A, Moradinia Z, Oraizi H, Keivaan A (2020). Bi-functional antenna coating for cloaking and directivity enhancement made of isotropic materials. Progress Electromagn. Res..

[66] Henrici P (1986). Applied and Computational Complex Analysis, Volume 3: Discrete Fourier Analysis, Cauchy Integrals, Construction of Conformal Maps, Univalent Functions.

[67] Yan W, Yan M, Ruan Z, Qiu M (2008). Coordinate transformations make perfect invisibility cloaks with arbitrary shape. New J. Phys..

[68] Zharova NA, Zharov AA, Zharov AA (2014). Conformal transformations in design of the coatings with gain-loss permittivity. Phys. Rev. A.

[69] Nehari Z (1952). Conformal Mapping, International Series in Pure and Applied Mathematics.

[70] Abramowitz M, Stegun IA (1965). Handbook of Mathematical Functions: With Formulas, Graphs, and Mathematical Tables. Dover Books on Mathematics.

[71] Inc., W. R. Mathematica, Version 12.1. Champaign, IL, 2020.

[72] Shilov GE, Silverman RA (1996). Elementary Real and Complex Analysis.

